# Supplementary material for: Identifying Individuals Who Currently Report Feelings of Anxiety Using Walking Gait and Quiet Balance: An Exploratory Study Using Machine Learning
Source: Sensors (Basel). 2022 Apr 20;22(9):3163. doi: 10.3390/s22093163 (PMC9105708; doi:10.3390/s22093163)
Supplement: Supplementary file 1 [file sensors-22-03163-s001.zip › sensors-1573544/Table S1.pdf]

Table S1 - Gait Variables

			Anxious		Not Anxious		
Variable	Relative Importance	Ranking	Mean	SD	Mean	SD	Significant Difference
Mean turns angle (°)	0.05	1	188.30	4.17	185.87	3.43	Yes
Variance neck bending in frontal plane (°)	0.03	2	1.74	1.18	2.04	1.14	
Variance in L arm swing velocity (°/s)	0.03	3	45.21	45.87	53.35	37.50	
Mean lumbar max. in sagittal plane (°)	0.02	4	5.46	5.10	3.27	4.42	Yes
Mean lumbar R rotation max. (°)	0.02	5	6.91	13.07	10.88	15.11	
Variance gait speed between legs (%)	0.02	7	0.91	0.80	1.23	0.74	Yes
Mean lumbar L bending max. in the frontal plane (°)	0.02	9	6.94	2.74	5.62	2.46	Yes
Variance neck R bending max. in the frontal plane (°)	0.02	10	2.31	1.58	2.49	1.25	
Variance step variability between legs (%)	0.02	12	8.16	5.85	11.21	7.44	Yes
Variance swing GCT between legs (s)	0.01	14	0.75	0.81	0.83	0.56	
Mean lower limb stance GCT (s)	0.01	15	60.52	1.51	59.94	1.48	Yes
Variance mid-swing elevation between legs (%)	0.01	17	13.88	11.97	18.98	13.94	Yes
Variance neck in the sagittal plane range (°)	0.01	18	3.31	2.01	3.77	1.37	Yes
Mean lumbar in the sagittal plane min. (°)	0.01	20	-0.66	5.02	-2.96	4.52	Yes
Variance stance of GCT between legs (%)	0.01	21	0.49	0.51	0.55	0.37	

Mean lumbar sagittal ROM (°)	0.01	22	5.22	1.41	5.46	1.49	
Avg. upper arm swing velocity (°/s)	0.01	23	195.99	85.54	186.72	55.98	
Variance GCD between legs (%)	0.01	24	0.23	0.22	0.15	0.25	
Mean lumbar rotational range in frontal plane (°)	0.01	27	11.67	4.77	10.91	2.79	
Mean lumbar L max. rot. (°)	0.01	29	4.76	12.43	0.03	15.23	Yes
Mean neck min. in sagittal plane (°)	0.01	31	-6.37	6.95	-7.90	6.84	
R Lower limb lateral step variability (cm)	0.01	34	2.74	0.80	2.72	0.63	
Variance foot strike angle between legs (%)	0.01	37	4.15	2.78	3.53	3.83	
Variance single support of GCT between legs (%)	0.01	38	0.81	0.76	0.83	0.55	
Variance neck rot. range in frontal plane (°)	0.01	41	2.78	1.74	3.80	2.95	Yes
Mean lumbar bending range in frontal plane (°)	0.01	42	9.82	3.12	8.78	3.16	Yes
Variance R lower limb terminal double support (% GCT)	0.01	44	0.84	0.33	0.76	0.15	Yes
Variance toe out angle between legs (%)	0.01	45	2.78	1.97	2.21	1.70	Yes
Variance neck R max. rot. (°)	0.01	46	12.78	17.13	9.55	5.61	Yes
Variance lumbar max. in sagittal plane (°)	0.01	47	1.52	0.61	1.62	0.63	
Variance L lower limb double support (% GCT)	0.01	48	1.10	0.39	1.15	0.31	
Mean trunk ROM in sagittal plane (°)	0.01	49	5.41	1.18	5.55	1.48	

Variance L arm ROM (°)	0.01	51	7.83	4.76	8.27	6.82	
Mean R lower limb elevation mid swing (cm)	0.01	53	1.43	0.71	1.40	0.83	
Mean L leg stride length (m)	0.00	54	1.20	0.09	1.23	0.12	
Mean trunk transverse ROM (°)	0.00	55	9.25	2.67	9.48	2.52	
Variance R leg circumduction (cm)	0.00	56	1.11	0.27	1.14	0.36	
Mean turns duration (#)	0.00	58	2.17	0.19	2.23	0.18	Yes
Lower limb # of contact points	0.00	60	25.07	5.73	23.84	5.32	
Mean R leg stride length (m)	0.00	61	1.18	0.08	1.20	0.12	
Variances double leg support GCT (%)	0.00	62	0.66	0.58	0.70	0.47	
Mean neck max. L bend in frontal plane (°)	0.00	63	2.07	3.40	2.28	3.36	
Mean # steps in turn	0.00	64	3.48	0.31	3.54	0.33	
Mean L arm swing velocity (°/s)	0.00	65	214.30	99.26	202.74	57.12	
Variance lumbar max. bend in frontal plane (°)	0.00	66	1.40	0.58	1.34	0.55	
Variance trunk coronal ROM (°)	0.00	67	1.02	0.42	1.10	0.58	
Variance R leg cadence steps (steps/min)	0.00	68	2.45	1.25	2.45	0.62	
Mean lumbar range in sagittal plane (°)	0.00	69	6.11	2.08	6.23	2.20	
Mean R arm ROM (°)	0.00	73	39.86	20.75	39.06	15.20	
Variance R arm swing velocity (°/s)	0.00	74	35.59	24.21	43.47	35.33	

Variance L foot strike angle (°)	0.00	76	1.78	0.61	1.81	0.51	
Mean leg double support RGCT	0.00	77	20.81	2.68	20.09	2.90	
Avg. gait speed	0.00	78	1.06	0.10	1.06	0.12	
Avg. circumduction	0.00	79	3.01	0.95	3.06	1.25	
Variance L toe out angle (°)	0.00	80	3.21	0.92	3.07	0.90	
Variance L leg elevation at mid. swing (cm)	0.00	81	0.41	0.13	0.40	0.12	
Mean lumbar coronal ROM (°)	0.00	82	6.54	2.79	5.72	1.86	Yes
Variance R arm ROM (°)	0.00	83	7.69	4.58	7.90	7.64	
Variance L leg stride length	0.00	84	0.04	0.01	0.04	0.01	
Variance L toe off angle (°)	0.00	85	1.45	0.57	1.40	0.53	
Variance neck max. in sagittal plane (°)	0.00	86	5.79	4.39	5.98	2.53	
Variance R leg double support RGCT	0.00	87	1.16	0.43	1.12	0.26	
Mean R toe off angle (°)	0.00	88	36.61	2.77	36.64	3.66	
Mean L leg GCD	0.00	90	1.14	0.08	1.16	0.09	
Mean lumbar transverse ROM	0.00	91	8.88	3.63	8.23	2.18	
Variance turns angle (°)	0.00	92	5.92	1.88	5.17	1.29	Yes
Variance avg. toe out angle between legs	0.00	93	863.54	4005.66	555.40	2466.91	
Variance L leg single limb support LGCT	0.00	96	0.73	0.28	0.81	0.39	

Mean neck R max. bending in frontal plane (°)	0.00	97	3.05	2.80	3.37	2.82	
Variance neck L max. bending in frontal plane (°)	0.00	98	2.32	1.69	2.36	1.07	
Avg. mid. swing elevation	0.00	102	1.41	0.57	1.33	0.66	
Mean R leg single limb support RGCT	0.00	103	39.72	1.27	39.85	1.53	
Mean step variability	0.00	104	1.41	0.57	1.33	0.66	
Mean L arm ROM (°)	0.00	105	47.47	21.40	44.17	14.99	
Variance neck in sagittal plane	0.00	109	5.80	3.97	5.76	2.26	
Variance R foot strike angle (°)	0.00	111	1.92	0.65	1.95	0.57	
Mean swing of GCT	0.00	115	39.66	1.37	40.03	1.47	
Variance steps in turn (#)	0.00	119	0.51	0.12	0.54	0.12	
Variance L leg speed	0.00	121	0.05	0.02	0.05	0.02	
Variance turn velocity (°)	0.00	125	22.22	8.45	21.50	8.14	
Mean toe out angle	0.00	126	36.81	2.64	36.78	3.62	
Variance lumbar min in the sagittal plane (°)	0.00	127	1.63	0.76	1.68	0.67	
Variance R leg support GCT	0.00	128	0.82	0.28	0.77	0.16	
Variance terminal double leg support of GCT	0.00	129	3.62	2.87	3.00	2.60	
Variance R toe off angle (°)	0.00	131	1.36	0.36	1.38	0.43	
Mean leg circumduction RCM	0.00	132	2.48	0.83	2.58	1.11	

Mean L leg stance GCT	0.00	133	60.16	1.33	60.01	1.56	
Mean neck R L rotation range (°)	0.00	137	7.39	2.46	8.25	2.44	Yes
Mean L foot strike angle (°)	0.00	138	24.49	3.48	24.37	4.53	
Variance R toe out angle (°)	0.00	139	3.19	0.92	3.04	0.87	
Variance lumbar coronal plane ROM (°)	0.00	142	0.76	0.32	0.73	0.25	
Variance lumbar R L range in the frontal plane (%)	0.00	146	1.25	0.50	1.28	0.57	
Mean neck L rotation max (°)	0.00	147	-10.32	17.77	-8.01	20.60	
Mean R leg swing (% GCT)	0.00	149	39.48	1.51	40.06	1.48	Yes
Mean L leg double support (% GCT)	0.00	155	20.57	2.70	19.91	2.91	
Mean R leg toe out angle (°)	0.00	156	6.78	6.77	5.70	7.07	
Variance R L lumbar rotation range (°)	0.00	157	1.57	0.49	1.73	0.88	
Variance R leg stance (% GCT)	0.00	158	0.75	0.27	0.72	0.25	
Mean trunk coronal ROM (°)	0.00	160	4.85	1.96	4.86	2.07	
Variance circumduction (%)	0.00	162	19.00	12.21	16.96	11.47	
Mean stride length (m)	0.00	163	1.19	0.08	1.21	0.12	
L leg lateral step variability (cm)	0.00	164	2.89	0.68	3.08	0.87	
Variance lumbar sagittal ROM (°)	0.00	167	0.92	0.26	1.02	0.38	
Mean cadence (steps/min)	0.00	171	105.91	7.73	104.42	7.98	

Mean R lumbar max in the frontal plane (°)	0.00	173	2.88	2.84	3.16	2.24	
Mean turn velocity (°/s)	0.00	175	184.22	26.74	178.97	20.32	
Variance in R leg swing (% GCT)	0.00	177	0.75	0.27	0.72	0.25	
Avg. foot strike angle (°)	0.00	178	24.59	3.67	24.34	4.55	
Mean L Leg single limb support (% GCT)	0.00	179	39.59	1.50	40.10	1.45	Yes
Variance L leg circumduction (cm)	0.00	183	1.25	0.46	1.25	0.41	
Variance in turn duration (s)	0.00	189	0.21	0.09	0.24	0.08	Yes
Variance in lumbar ROM in transverse place (°)	0.00	191	1.69	0.51	1.74	0.59	
Variance in L leg cadence (steps/min)	0.00	192	2.74	1.50	2.51	0.76	
Variance in lumbar L max. rot. °	0.00	193	5.16	4.18	5.89	6.67	
Variance in L leg swing (% GCT)	0.00	194	0.77	0.26	0.84	0.28	
Avg. double leg support (% GCT)	0.00	196	20.69	2.69	20.00	2.90	
Variance in R leg elevation mid. swing (cm)	0.00	198	0.52	0.25	0.60	0.38	
Mean R arm swing velocity (°/s)	0.00	200	177.68	78.69	170.70	60.36	
Variance in upper arm ROM between arms (%)	0.00	201	14.38	12.96	13.75	11.20	
Mean R foot strike angle (°)	0.00	202	24.69	4.22	24.32	4.83	
Mean neck max. in sagittal plane (°)	0.00	203	1.50	6.04	1.05	6.60	
Mean neck range in the sagittal plane (°)	0.00	205	7.88	2.67	8.96	2.96	Yes

Variance in step duration between legs (%)	0.00	207	0.87	0.75	0.83	0.65	
Variance in upper arm swing velocity between arms (%)	0.00	209	11.87	9.13	11.65	9.44	
Variance in R leg step duration (s)	0.00	210	0.02	0.01	0.02	0.01	
Variance in stride length between legs (m)	0.00	211	0.86	0.70	1.06	0.68	
Variance in L leg stance GCT (s)	0.00	212	0.77	0.26	0.84	0.28	
Variance in lumbar R max. rot. (°)	0.00	214	5.24	4.44	6.11	6.56	
Variance L leg terminal double support (% GCT)	0.00	216	0.71	0.16	0.76	0.27	
Variance R leg stride length (m)	0.00	218	0.04	0.01	0.04	0.01	
Avg. terminal double leg support (% GCT)	0.00	219	10.42	1.33	10.06	1.45	
Mean L leg gait speed (m/s)	0.00	220	1.06	0.11	1.07	0.12	
Mean R leg (% of GCD)	0.00	221	1.14	0.08	1.16	0.09	
Mean L leg swing (% GCT)	0.00	224	39.84	1.33	39.99	1.56	
Variance in R leg gait speed (m/s)	0.00	228	0.05	0.02	0.05	0.02	
Avg. GCD (d)	0.00	233	1.14	0.08	1.16	0.09	
Avg. single support (% GCT)	0.00	234	39.65	1.32	39.97	1.44	
Avg. stance (% GCT)	0.00	235	60.34	1.37	59.97	1.47	
Avg. step duration (s)	0.00	236	0.57	0.04	0.58	0.04	
Avg. upper arm ROM (°)	0.00	238	43.67	19.88	41.61	13.84	

Variance in lumbar range in sagittal plane (°)	0.00	241	1.28	0.52	1.31	0.55	
Variance in lumbar R max. bend in frontal plane (°)	0.00	242	1.21	0.66	1.18	0.50	
Variance in neck L max. rot. (°)	0.00	243	12.90	17.14	9.63	5.82	Yes
Mean neck R max. rot. (°)	0.00	244	17.71	17.18	16.26	21.44	
Mean neck bend range in frontal plane (°)	0.00	245	5.12	2.01	5.64	2.05	
Mean L leg cadence (steps/min)	0.00	246	106.08	7.78	104.61	8.05	
Mean R leg cadence (steps/min)	0.00	247	105.73	7.69	104.23	7.91	
Mean L leg circumduction (cm)	0.00	248	3.54	1.27	3.54	1.54	
Mean L leg elevation mid. swing (cm)	0.00	249	1.38	0.53	1.27	0.64	
Variance L leg GCD (s)	0.00	250	0.03	0.02	0.03	0.01	
Variance R leg GCD (s)	0.00	251	0.03	0.02	0.03	0.01	
Mean R leg gait speed (m/s)	0.00	252	1.05	0.10	1.05	0.12	
Mean L leg step duration (s)	0.00	253	0.57	0.04	0.58	0.05	
Variance in L leg step duration (s)	0.00	254	0.02	0.01	0.02	0.01	
Mean R leg step duration (s)	0.00	255	0.57	0.04	0.58	0.04	
Mean L leg terminal double support (% GCT)	0.00	256	10.26	1.47	10.12	1.59	
Mean R leg terminal double support (% GCT))	0.00	257	10.58	1.34	10.01	1.41	Yes
Mean L leg toe off angle (°)	0.00	258	37.01	3.07	36.93	3.86	

Mean L leg toe out angle (°)	0.00	259	2.19	6.23	2.17	7.34	
Variance in trunk ROM in sagittal plane (°)	0.00	260	1.06	0.35	1.19	0.43	Yes
Variance in trunk ROM in transverse plane (°)	0.00	261	1.81	0.58	2.05	1.18	Yes

L = left, R = right, rot = rotation, ROM = range of motion, max. = maximum, min. = minimum, avg. = average, GCT = gait cycle time, # = number, % = percentage, cm= centimeter, GCD = gait cycle duration, ° = degrees
