# Supplementary material for: Identifying Individuals Who Currently Report Feelings of Anxiety Using Walking Gait and Quiet Balance: An Exploratory Study Using Machine Learning
Source: Sensors (Basel). 2022 Apr 20;22(9):3163. doi: 10.3390/s22093163 (PMC9105708; doi:10.3390/s22093163)
Supplement: Supplementary file 1 [file sensors-22-03163-s001.zip › sensors-1573544/Anxiety Balance Table S2-S5.pdf]

Table S2 - Eyes Open, Feet on Ground

			Anxious		Not Anxious		
Variable	Relative Importance	Ranking	Mean	SD	Mean	SD	Significant Difference
Acceleration path length in sagittal plane (m/s^2)	0.01	26	4.17	2.04	3.97	2.14	
Frequency dispersion in coronal plane	0.01	30	0.65	0.06	0.67	0.06	
Centroidal frequency in coronal plane (Hz)	0.01	33	1.32	0.27	1.23	0.37	
Centroidal frequency (Hz)	0.01	35	0.89	0.22	0.95	0.20	
Mean velocity in sagittal plane (m/s)	0.01	39	0.18	0.26	0.11	0.08	Yes
Jerk (m^2/s^5)	0.00	59	1.49	2.66	1.36	2.48	
Sway angle area (°)	0.00	95	1.24	2.31	0.86	0.93	Yes
RMS sway angle (°)	0.00	100	0.52	0.62	0.37	0.20	Yes
Frequency dispersion	0.00	118	0.68	0.05	0.69	0.05	
Mean velocity (m/s)	0.00	141	0.19	0.26	0.12	0.10	Yes
Jerk in coronal plane (m^2/s^5)	0.00	144	0.41	0.34	0.57	0.82	Yes
Mean velocity in coronal plane (m/s)	0.00	150	0.03	0.02	0.04	0.05	
Acceleration 95% ellipse radius on y-axis (m/s^2)	0.00	153	0.21	0.25	0.15	0.08	Yes
Sway angle area radius in coronal plane (°)	0.00	182	0.23	0.10	0.27	0.13	
Acceleration 95% ellipse rot on y-axis (m/s^2)	0.00	187	1.59	0.13	1.56	0.21	

Angle 95% ellipse radius on y-axis (°)	0.00	188	1.25	1.52	0.87	0.49	Yes
Jerk in sagittal plane (m^2/s^5)	0.00	215	1.71	3.09	1.56	2.86	
Angle RMS Sway in sagittal plane (°)	0.00	217	0.51	0.62	0.34	0.18	Yes
Acceleration 95% ellipse radius on x-axis (m/s^2)	0.00	262	0.04	0.02	0.05	0.02	
Acceleration 95% ellipse sway area (m^2/s^4)	0.00	265	0.04	0.07	0.02	0.03	Yes
Acceleration path length in coronal plane (m/s^2)	0.00	277	2.52	0.96	2.65	1.46	
Acceleration path length (m/s^2)	0.00	280	5.37	2.31	5.28	2.72	
Acceleration range in coronal plane (m/s^2)	0.00	283	0.11	0.06	0.13	0.09	Yes
Acceleration range (m/s^2)	0.00	284	0.47	0.63	0.35	0.26	Yes
Acceleration range in sagittal plane (m/s^2)	0.00	288	0.45	0.63	0.32	0.25	Yes
Acceleration RMS sway in coronal plane (m/s^2)	0.00	292	0.02	0.01	0.02	0.02	Yes
Acceleration RMS sway (m/s^2)	0.00	293	0.09	0.10	0.06	0.03	Yes
Acceleration RMS sway in the sagittal plane (m/s^2)	0.00	295	0.09	0.10	0.06	0.03	Yes
Angle Durations (s)	0.00	300	29.99	0.00	29.99	0.00	Yes
Angle RMS sway in coronal plane (°)	0.00	302	0.11	0.05	0.13	0.11	Yes
Angle sway area rot (°)	0.00	309	1.59	0.13	1.56	0.21	

rot = rotation, RMS=Root mean square, ° = degrees, $^{\circ 2}$ = degrees^2

Table S3 - Eyes Closed, Feet on Ground

			Anxious		Not Anxious		
Variable	Relative Importance	Ranking	Mean	SD	Mean	SD	Significant Difference
Frequency dispersion	0.01	28	0.66	0.06	0.66	0.04	
Acceleration 95% ellipse rot (m/s^2)	0.01	32	1.55	0.14	1.62	0.25	Yes
Centroidal frequency (Hz)	0.01	52	0.93	0.24	0.95	0.21	
Acceleration 95% ellipse radius on y-axis (m/s^2)	0.00	107	0.16	0.06	0.16	0.04	
Frequency dispersion in coronal plane	0.00	112	0.66	0.06	0.67	0.06	
Acceleration RMS sway (m/s^2)	0.00	114	0.07	0.02	0.07	0.02	
RMS sway angle (°)	0.00	120	0.39	0.15	0.40	0.10	
Frequency dispersion in sagittal plane	0.00	124	0.69	0.05	0.70	0.05	
Acceleration 95% ellipse radius on x-axis (m/s^2)	0.00	135	0.04	0.01	0.05	0.02	
Acceleration RMS sway in sagittal plane (m/s^2)	0.00	143	0.06	0.02	0.06	0.02	
Jerk in coronal plane (m^2/s^5)	0.00	148	0.37	0.28	0.54	0.67	Yes
Jerk (m^2/s^5)	0.00	159	0.99	0.70	1.22	0.98	
Sway area rot (°)	0.00	165	1.55	0.14	1.62	0.25	Yes
Acceleration path length in sagittal plane (m/s^2)	0.00	166	4.27	1.54	4.53	1.57	
Acceleration path length (m/s^2)	0.00	168	5.41	1.80	5.84	2.16	

Centroidal frequency in sagittal plane (Hz)	0.00	170	0.73	0.20	0.75	0.17	
Mean velocity in sagittal plane (m/s)	0.00	172	0.10	0.05	0.12	0.05	Yes
Angle durations (s)	0.00	174	29.99	0.00	29.99	0.00	Yes
Angle RMS in sagittal plane (°)	0.00	190	0.37	0.15	0.37	0.10	
Centroidal frequency in coronal plane (Hz)	0.00	195	1.21	0.23	1.20	0.32	
Acceleration RMS sway in coronal plane (m/s^2)	0.00	197	0.02	0.01	0.02	0.01	
Jerk in sagittal plane (m^2/s^5)	0.00	204	1.17	0.89	1.38	1.07	
Mean velocity in coronal plane (m/s)	0.00	222	0.03	0.02	0.03	0.03	
Angle sway area ($°^2$)	0.00	226	0.73	0.39	0.85	0.50	
Acceleration path length in coronal plane (m/s^2)	0.0	229	2.48	0.82	2.75	1.38	
Acceleration 95% ellipse sway area (m^2/s^4)	0.00	266	0.02	0.01	0.02	0.01	
Mean velocity (m/s)	0.00	274	0.11	0.06	0.13	0.05	
Acceleration range (m/s^2)	0.00	285	0.35	0.12	0.37	0.10	
Acceleration range in sagittal plane (m/s^2)	0.00	289	0.33	0.12	0.34	0.11	
Angle 95% ellipse radius in y-axis (°)	0.00	297	0.92	0.36	0.93	0.24	
Angle RMS sway in coronal plane (°)	0.00	303	0.11	0.04	0.12	0.07	

rot = rotation, RMS=Root mean square, ° = degrees, $°^2$ = degrees^2

Table S4 - Eyes Open, Feet on Foam Surface

			Anxious		Not Anxious		
Variable	Relative Importance	Ranking	Mean	SD	Mean	SD	Significant Difference
Mean velocity in coronal plane (m/s)	0.02	6	0.05	0.04	0.06	0.03	
Velocity range in coronal plane (m/s^2)	0.02	8	0.17	0.05	0.21	0.07	Yes
Frequency dispersion	0.01	16	0.66	0.06	0.68	0.04	Yes
Acceleration 95% ellipse radius on x-axis (m/s^2)	0.01	19	0.07	0.02	0.08	0.03	Yes
Centroidal frequency in coronal plane (Hz)	0.01	36	1.09	0.15	1.00	0.24	Yes
Sway angle area radius in coronal plane (°)	0.01	40	0.40	0.10	0.49	0.15	Yes
RMS sway angle in coronal plane °	0.01	43	0.18	0.05	0.21	0.07	Yes
Frequency dispersion in coronal plane	0.00	57	0.65	0.05	0.67	0.06	
Centroidal frequency (Hz)	0.00	106	1.02	0.19	1.03	0.22	
Acceleration RMS sway in sagittal plane (m/s^2)	0.00	108	0.07	0.03	0.07	0.02	
RMS sway angle (°)	0.00	116	0.43	0.15	0.45	0.14	
Frequency dispersion in sagittal plane	0.00	154	0.69	0.04	0.71	0.04	Yes
Acceleration RMS sway in coronal plane (m/s^2)	0.00	176	0.03	0.01	0.04	0.01	Yes
Jerk (m^2/s^5)	0.00	180	1.38	0.75	1.62	1.34	
Sway area rot (°)	0.00	184	1.58	0.28	1.52	0.34	

Mean velocity (m/s)	0.00	185	0.16	0.09	0.15	0.07	
Centroidal frequency in sagittal plane (Hz)	0.00	186	0.87	0.20	0.91	0.23	
Acceleration 95% ellipse radius on y-axis (m/s^2)	0.00	223	0.17	0.06	0.17	0.06	
Acceleration 95% ellipse rot (m/s^2)	0.00	264	1.58	0.28	1.52	0.34	
Acceleration 95% ellipse sway area (m^2/s^4)	0.00	267	0.04	0.02	0.05	0.03	Yes
Jerk in coronal plane (m^2/s^5)	0.00	271	0.78	0.47	0.99	0.82	Yes
Jerk in sagittal plane (m^2/s^5)	0.00	272	1.56	0.88	1.92	1.75	Yes
Mean velocity in sagittal plane (m/s)	0.00	276	0.14	0.09	0.13	0.07	
Acceleration path length in coronal plane (m/s^2)	0.00	278	3.56	1.01	3.86	1.46	
Acceleration path length (m/s^2)	0.00	281	6.79	1.70	7.22	2.65	
Acceleration path length in sagittal plane (m/s^2)	0.00	282	4.97	1.48	5.22	2.11	
Acceleration range (m/s^2)	0.00	286	0.39	0.12	0.41	0.13	Yes
Acceleration range in sagittal plane (m/s^2)	0.00	290	0.35	0.12	0.35	0.12	
Acceleration RMS sway (m/s^2)	0.00	294	0.07	0.03	0.08	0.02	
Angle 95% ellipse radius in y-axis (°)	0.00	298	0.97	0.38	0.98	0.33	Yes
Angle durations	0.00	301	29.99	0.00	29.99	0.00	
Angle RMS sway in sagittal plane (°)	0.00	305	0.39	0.16	0.39	0.14	Yes
Angle sway area ($°^2$)	0.00	306	1.27	0.64	1.59	0.95	Yes

rot = rotation, RMS=Root mean square, ° = degrees, $°^2$ = $degrees^2$

Table S5 - Eyes Closed, Feet on Foam Surface

			Anxious		Not Anxious		
Variable	Relative Importance	Ranking	Mean	SD	Mean	SD	Significant Difference
Acceleration 95% ellipse rot (m/s^2)	0.02	13	1.51	0.30	1.63	0.25	Yes
Acceleration path length in sagittal plane (m/s^2)	0.01	25	8.37	3.29	9.25	3.53	
Frequency dispersion	0.00	94	0.65	0.05	0.65	0.05	
Mean velocity in sagittal plane (m/s)	0.00	99	0.18	0.09	0.19	0.14	
Acceleration 95% ellipse radius on axis 1 (m/s^2)	0.00	110	0.13	0.04	0.13	0.04	
Mean velocity in coronal plane (m/s)	0.00	117	0.07	0.03	0.08	0.04	
Frequency dispersion in coronal plane	0.00	122	0.63	0.07	0.65	0.05	Yes
Rot sway area (°)	0.00	123	1.51	0.30	1.63	0.25	Yes
Centroidal frequency in coronal plane (Hz)	0.00	130	0.88	0.14	0.93	0.23	
Acceleration range in coronal plane (m/s^2)	0.00	134	0.34	0.13	0.34	0.14	
Acceleration RMS sway in coronal plane (m/s^2)	0.00	136	0.06	0.02	0.06	0.02	
Jerk in coronal plane (m^2/s^5)	0.00	140	2.09	1.31	2.93	3.24	Yes
Acceleration RMS sway (m/s^2)	0.00	145	0.12	0.03	0.12	0.04	
RMS sway angle in coronal plane °	0.00	169	0.33	0.11	0.34	0.11	
Jerk (m^2/s^5)	0.00	181	4.31	3.98	5.05	4.13	

Acceleration path length (m/s^2)	0.00	199	11.19	3.73	12.42	4.74	
Angle RMS sway in sagittal plane	0.00	206	0.60	0.18	0.63	0.24	
Centroidal frequency in sagittal plane (Hz)	0.00	225	0.84	0.21	0.83	0.17	
Angle durations	0.00	230	29.99	0.00	29.99	0.00	Yes
Acceleration 95% ellipse radius on y-axis (m/s^2)	0.00	263	0.25	0.08	0.27	0.10	
Acceleration 95% ellipse sway area (m^2/s^4)	0.00	268	0.11	0.06	0.12	0.07	
Centroidal frequency (Hz)	0.00	269	1.05	0.17	1.04	0.17	
Frequency dispersion in sagittal plane	0.00	270	0.69	0.05	0.69	0.05	
Jerk in sagittal plane (m^2/s^5)	0.00	273	5.16	5.13	5.79	4.63	
Mean velocity (m/s)	0.00	275	0.20	0.08	0.22	0.14	
Acceleration path length in coronal plane (m/s^2)	0.00	279	5.67	1.71	6.28	2.86	
Acceleration range (m/s^2)	0.00	287	0.68	0.24	0.69	0.24	
Acceleration range in sagittal plane (m/s^2)	0.00	291	0.58	0.22	0.59	0.22	
Acceleration RMS sway in sagittal plane (m/s^2)	0.00	296	0.10	0.03	0.11	0.04	Yes
Angle 95% ellipse radius in y-axis (°)	0.00	299	1.49	0.45	1.57	0.58	Yes
Angle RMS sway (°)	0.00	304	0.69	0.20	0.72	0.24	Yes
Angle sway area (^{2})	0.00	307	3.77	2.09	4.15	2.56	Yes
Angle sway area radius in coronal plane ($°^2$)	0.00	308	0.76	0.26	0.79	0.26	

rot = rotation, RMS=Root mean square, ° = degrees, $°^2$ = degrees^2
